# Opinions and knowledge about organ donation and transplantation: a multicenter cross-sectional study among medical science students in Poland

**DOI:** 10.3389/fpubh.2025.1664859

**Published:** 2025-11-14

**Authors:** Paulina Mularczyk-Tomczewska, Łukasz Czyżewski, Magdalena Łoś, Justyna Grudziąż-Sękowska, Janusz Wyzgał, Andrzej Silczuk

**Affiliations:** 1 Department of Public Health, Medical University of Warsaw, Warsaw, Poland; 2 Department of Geriatric Nursing, Medical University of Warsaw, Warsaw, Poland; 3 Department of Social Medicine and Public Health, Medical University of Warsaw, Warsaw, Poland; 4 School of Public Health, Centre of Postgraduate Medical Education, Warsaw, Poland; 5 Department of Nephrology Nursing, Medical University of Warsaw, Warsaw, Poland; 6 Department of Community Psychiatry, Medical University of Warsaw, Warsaw, Poland

**Keywords:** organ donation, transplantation, presumed consent, medical students, legal knowledge, health education

## Abstract

**Purpose:**

To assess attitudes toward organ procurement and transplantation, knowledge of legal regulations, and sources of information among medical students and students of other healthcare disciplines in Poland.

**Materials and methods:**

A multicenter cross-sectional survey was conducted among 3,006 students from four Polish medical universities. The study included 883 medical students (MD program) and 2,122 students of other healthcare-related programs. An original questionnaire was used to assess attitudes, legal knowledge, and sources of information. A transplantation support index, reflecting the overall level of pro-donation attitudes, was developed. Data were analyzed using descriptive and non-parametric statistical methods.

**Results:**

The median Transplantation Support Index (TSI) was higher among medical students [24.00 (IQR: 21.00–25.00; mean ± SD: 23.41 ± 3.02)] compared to students of other healthcare disciplines [22.00 (IQR: 20.00–24.00; mean ± SD: 21.87 ± 3.45); *p* < 0.001]. Most respondents supported organ transplantation from deceased donors (97%) and agreed to donate their own organs posthumously (91%). Only 49% of all students correctly identified the legal model of presumed consent in Poland—66% among medical students and 41% among others. The most common sources of knowledge were academic classes (63%) and the Internet (62%).

**Conclusion:**

Students of medical universities in Poland generally support organ transplantation. However, significant gaps in legal knowledge exist, especially among students of other healthcare disciplines. These findings highlight the need to strengthen and standardize transplantation-related education in healthcare curricula to foster pro-donation attitudes and increase awareness of legal frameworks.

## Introduction

Organ donation and transplantation are not only advanced medical procedures but also critical components of public health systems, reflecting the efficiency of healthcare infrastructure, the effectiveness of health education, and the ethical and social cohesion of modern societies. Addressing barriers to organ donation is therefore a key public health priority, particularly in countries with significant gaps between supply and demand (1). Although organ transplantation is an increasingly common procedure, the main factor limiting its use is the shortage of available organs (2). The field of organ donation and transplantation is now highly regulated in many countries. The legal framework defining the standards for organ transplantation is set out in Directive 2010/53/EU and Commission Implementing Directive 2012/25/EU lays down the information procedures for the exchange, between EU member states, of human organs intended for transplantation (3, 4). Both directives should already be transposed into the national legislations of the EU member states (5). The directive lay down rules to ensure quality and safety standards in organ transplantation. In addition, it aims to ensure that donors and recipients have the same quality, safety and legal standards regardless of where they live. The scope of the Directive covers issues related to organ donation, testing and characterization, organ procurement and transplantation (3).

Currently, the main source of organs for transplantation in Europe is from deceased donors, with donations from brain-dead donors overtaking those from circulatory death donors. According to the European Commission’s 2017 study on the use and impact of the EU Action Plan on Organ Donation and Transplantation (2009–2015) in EU Member States, deceased donation is the source for kidney, liver, heart, lung, pancreas and small bowel transplants. Living donation is mainly used for kidney transplants and some liver transplants (6). The insufficient number of organs and increasing demand is currently a problem throughout Europe. Organ donation rates for both deceased and living donors vary widely across EU countries. In recent decades, Spain has been widely considered a global benchmark in organ donation and transplantation. For example, Matesanz et al. (7) describe how Spain achieved ~40 deceased donors per million population via systemic changes in donor detection, hospital coordination, and public awareness campaigns. Moreover, in 2019 Spain reached a historic peak of 49 donors per million population and more than 5,400 transplant procedures (8). These results illustrate how structural and organizational policies can substantially shift donation rates, and provide a useful comparative model for countries seeking to optimize their systems.

According to the Global Observatory on Donation and Transplantation, significant disparities in organ donation rates persist across countries, despite similar legislative frameworks (2). For instance, in 2023, Spain—a global leader in organ transplantation—reported over 46 deceased donors per million population (pmp), followed by countries such as Portugal, France, and Croatia, all exceeding 30 pmp (5). In contrast, Poland reported approximately 12–13 deceased donors pmp, placing it well below the EU average (9). These differences underscore that legal models alone (e.g., opt-out systems) do not guarantee high donation rates (10). Rather, public awareness, cultural attitudes, healthcare infrastructure, and professional training appear to be critical factors in determining transplant success. In recent years, the European Commission and global health bodies have increasingly emphasized the importance of medical education in shaping pro-donation attitudes and improving donation rates. Public trust and willingness to donate organs are strongly influenced by the knowledge, communication skills, and personal attitudes of healthcare professionals—beginning already at the university level (11).

There are different systems in EU Member States that allow people to consent to organ donation after death. In an explicit consent system, also known as “opt-in,” consent must be given explicitly during life. The implied consent system, also called “opt-out,” is characterized by the principle of “presumed consent” (silence is equivalent to consent). However, it is possible not to consent to organ removal after death by making an objection. There are also mixed systems (5). The opt-out system is often considered to be a contributing factor to higher donation rates. Increasing organ donation by adopting an opt-out system is widely discussed among the public (12). In this context, a recent study comparing opt-in and opt-out systems in 35 similar countries registered with the Organization for Economic Co-operation and Development (OECD) found no significant difference in deceased donor rates; however, a reduction in living donors was observed in opt-out countries. Of 35 OECD-registered countries, 17 are classified as opt-out (or presumed consent) countries and 18 classed as opt-in countries (8). The authors suggest that “other barriers to organ donation need to be addressed, even in countries where consent for donation is presumed,” and conclude that “greater emphasis on educating and informing the general public about the benefits of transplantation is the preferred way to achieve an increase in organ donation” (8).

The Polish Trasplantation Act of 1 July 2005 regulates the collection, storage and transplantation of cells, tissues and organs (13). It implements the provisions of the European Union law, particularly Directive 2004/23/EC (14). The Polish transplantation act specifies the rules of procurement, storage and transplantation of cells, including haematopoietic cells from marrow, peripheral blood, cord blood and tissues and organs derived from living or deceased donors, as well as the rules of testing, processing, storage and distribution of human cells and tissues (13). According to article 1, this Act does not apply to the collection, transplantation of reproductive cells, gonads, embryonic and fetal tissues, reproductive organs and parts thereof, and does not regulate the collection, storage and distribution of blood for transfusion, separation of its components or processing into drugs (13, 15). The act specifies for what purposes cells, tissues and organs may be collected. These are diagnostic, therapeutic, scientific and didactic purposes. In Poland, according to Article 5 of the Transplantation Act, the model of presumed consent is applied, which means that organs may be taken from the corpse, if the deceased did not object during his/her lifetime. Importantly, the Polish Transplantation Act does not stipulate that the family of a deceased person should consent to the removal of organs. They do not have the right to dispose of the remains, but only the right to burial (13, 16). According to data from the Polish Transplant Coordination Center “Poltransplant” in 2024 a total of 1,538 organ transplantations were performed in Poland. This included 1,136 kidney transplants (of which 61 were from living donors), 243 liver transplants, 89 heart transplants, 60 lung transplants, and 10 pancreas transplants. That same year, 1,210 potential deceased donors were reported, yet organ retrieval was carried out in only 455 cases. Moreover, the number of individuals officially registered as objecting to post-mortem organ donation in the Central Register of Objections amounted to 47,391 (7). Given that organ donation involves not only clinical but also societal processes—including population-level education, trust in the health system, and equity in access—public health approaches must complement legal and institutional frameworks. Effective donor strategies require sustained community engagement, clear communication policies, and inclusion of vulnerable populations. Therefore, it becomes essential to assess the perspectives of future healthcare professionals on this issue. Understanding their level of knowledge and attitude toward transplantation can provide a basis for targeted educational and policy strategies aimed at closing the gap between legal frameworks and actual donation rates. Recent evidence from multiple regions confirms persistent educational gaps and cross-cultural variability in medical students’ knowledge and attitudes toward organ donation, underscoring the need for structured, evidence-based curricula (17–22).

### Purpose

The purpose of this study was to assess: (1) medical students’ attitudes toward organ procurement and transplantation; (2) their knowledge of current legal regulations regarding organ donation; and (3) the sources of their knowledge on organ procurement and transplantation in Poland. In addition, this study sought to explore whether differences in knowledge and attitudes toward organ donation and transplantation may coexist within the same educational environment and between distinct academic tracks. By comparing medical students with those enrolled in other healthcare disciplines, the study aimed to provide empirical insight into how educational exposure and curricular content may shape students’ understanding of transplantation law and their readiness to support or discuss organ donation.

## Materials and methods

### Study design and population

A multicenter cross-sectional survey was conducted between 2017 and 2019 among medical students of the Medical University in Białystok, Karol Marcinkowski Medical University in Poznań, Collegium Medicum of the Jagiellonian University and Medical University of Warsaw (Poland). The student population consisted of 3,006 students, including 883 students of medicine (MD program), referred to as the MD student group. The remaining 2,122 students were enrolled in other healthcare—related programs—such as nursing, midwifery, emergency medical services, and public health and were classified as students of other healthcare disciplines. All questionnaires were printed and delivered to each of the subjects personally by a member of the research team. Questionnaires were answered by students during breaks between classes or lectures. Participation in the study was voluntary and anonymous. Participants had the right to refuse to participate without giving a reason. The study protocol was reviewed and approved by the Ethical Review Board at the Medical University of Warsaw, Poland [decision number: (AUBE/81/16)]. The questionnaire was anonymous, so it was not possible to agree to participate in writing. The letter of invitation contained information that the fulfilment and return of the questionnaire implied an implicit consent to participate in the survey (see Tables 1–8).

**Table 1:** Study group characteristics.

Characteristics	Students of medicine		Other healthcare students		*p*	Strength of effect	Test *post-hoc* (*p*)
	*N*	*n* (%)	*N*	*n* (%)			
Sex							
Female	883	557 (63.1)	2,122	1,899 (89.5)	<0.001	ϕ = 0.05	nd
Male		326 (36.9)		223 (10.5)			
Age, years							
Median (*Q1*–*Q3*)	882	21.00 (20.00–24.00)	2,119	22.00 (21.00–23.00)	<0.001	*r_g_* = −0.14	nd
Place of residence							
Village	883	163 (18.5)	2,122	695 (32.8)	<0.001	*V* = 0.18	<0.001
City <5 thousand inhabitants		20 (2.3)		90 (4.2)			0.093
City with 5–10 thousand inhabitants		31 (3.5)		97 (4.6)			>0.999
City 10–20 thousand inhabitants		62 (7.0)		135 (6.4)			>0.999
City 20–50 thousand inhabitants		121 (13.7)		248 (11.7)			>0.999
Town 50–100 thousand inhabitants		110 (12.5)		185 (8.7)			0.016
City with 100–200 thousand residents		91 (10.3)		107 (5.0)			<0.001
City >200 thousand inhabitants		285 (32.3)		565 (26.6)			0.016
Mother’s education							
Primary	878	20 (2.3)	2,115	149 (7.0)	<0.001	*V* = 0,31	<0.001
Secondary		190 (21.6)		975 (46.1)			<0.001
Higher		656 (74.7)		874 (41.3)			<0.001
Not applicable		12 (1.4)		117 (5.5)			<0.001
Father’s education							
Primary	879	34 (3.9)	2,102	251 (11.9)	<0.001	*V* = 0,38	<0.001
Secondary		248 (28.2)		1.186 (56.4)			<0.001
Higher		581 (66.1)		553 (26.3)			<0.001
Not applicable		16 (1.8)		112 (5.3)			<0.001
Material situation							
Very good	882	36 (4.1)	2,120	65 (3.1)	<0.001	*V* = 0,15	0.783
Good		218 (24.7)		645 (30.4)			0.007
Satisfactory		392 (44.4)		1.108 (52.3)			<0.001
Bad		236 (26.8)		302 (14.2)			<0.001

ϕ (phi coefficient) is a measure of effect size for 2 × 2 contingency tables, with 0.1, 0.3, and 0.5 indicating small, medium, and large effects, respectively.

Cramer’s *V* is used for contingency tables larger than 2 × 2, with similar thresholds of interpretation.

rg (rank-biserial correlation) is the effect size for the Mann–Whitney *U* test, where values closer to ±1 indicate stronger associations.

**Table 2:** Detailed structure of responses to the component questions of the transplantation support index of the whole study group.

Question	Answer				
Whole study group	1—definitely no	2	3	4	5—definitely yes
Do you support the removal and transplantation of organs from deceased donors?	15 (0.5)	26 (0.9)	64 (2.1)	514 (17.1)	2,388 (79.4)
Would you agree to your organs being taken in the event of your death?	30 (1.0)	56 (1.9)	188 (6.3)	750 (24.9)	1983 (65.9)
Would you accept your loved one’s organs being taken from you after your death?	34 (1.1)	87 (2.9)	429 (14.3)	1,063 (35.4)	1,392 (46.3)
Do you support the removal of organs from living donors?	56 (1.9)	128 (4.3)	413 (13.7)	1,223 (40.7)	1,187 (39.5)
Is organ transplantation compatible with your religion or ethical views?	71 (2.4)	88 (2.9)	392 (13.0)	757 (25.2)	1,699 (56.5)

**Table 3:** Detailed structure of responses to the component questions of the transplantation support index of students of medicine.

Question	Answer				
Students of medicine	1—definitely no	2	3	4	5—definitely yes
Do you support the removal and transplantation of organs from deceased donors?	6 (0.7)	11 (1.2)	7 (0.8)	91 (10.3)	770 (87.0)
Would you agree to your organs being taken in the event of your death?	15 (1.7)	15 (1.7)	34 (3.8)	178 (20.1)	643 (72.7)
Would you accept your loved one’s organs being taken from you after your death?	11 (1.2)	16 (1.8)	79 (8.9)	257 (29.0)	522 (59.0)
Do you support the removal of organs from living donors?	17 (1.9)	25 (2.8)	86 (9.7)	309 (34.9)	448 (50.6)
Is organ transplantation compatible with your religion or ethical views?	23 (2.6)	21 (2.4)	58 (6.6)	167 (18.9)	616 (69.6)

**Table 4:** Detailed structure of responses to the component questions of the transplantation support index of the students of other healthcare disciplines.

Question	Answer				
Students of other healthcare disciplines	1—definitely no	2	3	4	5—definitely yes
Do you support the removal and transplantation of organs from deceased donors?	9 (0.4)	15 (0.7)	57 (2.7)	423 (19.9)	1,618 (76.2)
Would you agree to your organs being taken in the event of your death?	15 (0.7)	41 (1.9)	154 (7.3)	572 (27.0)	1,340 (63.1)
Would you accept your loved one’s organs being taken from you after your death?	23 (1.1)	71 (3.3)	350 (16.5)	806 (38.0)	870 (41.0)
Do you support the removal of organs from living donors?	39 (1.8)	103 (4.9)	327 (15.4)	914 (43.1)	739 (34.8)
Is organ transplantation compatible with your religion or ethical views?	48 (2.3)	67 (3.2)	334 (15.7)	590 (27.8)	1,083 (51.0)

**Table 5:** Comparison of transplantation support index levels between students of medicine and students of other healthcare disciplines.

Question	Students of medicine	Students of other healthcare disciplines	*p*	Strength of effect (*r_g_*)
Do you support the removal and transplantation of organs from deceased donors?	5.00 (5.00–5.00)	5.00 (5.00–5.00)	<0.001	0.12
Would you agree to your organs being taken in the event of your death?	5.00 (4.00–5,00)	5.00 (4.00–5.00)	<0.001	0.09
Would you accept your loved one’s organs being taken from you after your death?	5.00 (4.00–5,00)	4.00 (4.00–5.00)	<0.001	0.17
Do you support the removal of organs from living donors?	5.00 (4.00–5,00)	4.00 (4.00–5.00)	<0.001	0.15
Is organ transplantation compatible with your religion or ethical views?	5.00 (4.00–5,00)	5.00 (4.00–5.00)	<0.001	0.17
Transplantation support index	24.00 (21.00–25.00)	22.00 (20.00–24.00)	<0.001	0.20

**Table 6:** Comparison of the percentage of people undecided about transplantation between students of medicine and students of other healthcare disciplines.

Question	Students of medicine	Students of other healthcare disciplines	*p*	Strength of effect *(ϕ)*
Do you support the removal and transplantation of organs from deceased donors?	7 (0.8)	57 (2.7)	0.002	0.06
Would you agree to your organs being taken in the event of your death?	34 (3.8)	154 (7.3)	0.001	0.06
Would you accept your loved one’s organs being taken from you after your death?	79 (8.9)	350 (16.5)	<0.001	0.10
Do you support the removal of organs from living donors?	86 (9.7)	327 (15.4)	<0.001	0.08
Is organ transplantation compatible with your religion or ethical views?	58 (6.6)	334 (15.7)	<0.001	0.12

**Table 7:** Comparison of knowledge of legal regulations concerning organ transplantation in Poland between students of medicine and students of other healthcare disciplines.

Knowledge of legal regulations concerning organ transplantation	Students of medicine	Students of other healthcare disciplines	*p*	Strength of effect *V*	Test *post-hoc* (*p*)
Correct answer	586 (66.3)	874 (41.2)	<0.001	0.23	<0.001
Wrong answer	254 (28.7)	1,096 (51.7)			<0.001
I do not know	44 (5.0)	151 (7.1)			0.185
The consent of the deceased while alive is needed	97 (11.0)	590 (27.8)	<0.001	0.25	<0.001
No objection from the deceased while alive is sufficient	586 (66.3)	874 (41.2)			<0.001
Consent of the family of the deceased is required	130 (14.7)	469 (22.1)			<0.001
No legal regulations in Poland	27 (3.1)	37 (1.7)			0.165
I do not know	44 (5.0)	151 (7.1)			0.185

**Table 8:** Comparison of sources of organ transplantation knowledge acquisition between students of medicine and students of other healthcare disciplines.

Sources of knowledge about organ transplantation	Students of medicine	Students of other healthcare disciplines	*p*	Strength of effect *ϕ*
Academic classes	553 (62.5)	1,330 (62.7)	>0.999	<0.01
Television	266 (30.1)	801 (37.7)	<0.001	0.07
Internet	495 (55.9)	1,362 (64.2)	<0.001	0.08
Books	170 (19.2)	396 (18.7)	0.744	0.01
Newspapers	126 (14.2)	290 (13.7)	0.705	0.01
Colleagues	130 (14.7)	313 (14.8)	>0.999	<0.01
Radio	61 (6.9)	140 (6.6)	0.818	0.01
Other source	166 (18.8)	353 (16.6)	0.169	0.03

### Study questionnaire

The questionnaire was purpose-designed for this study to assess knowledge, attitudes, and declared behaviors related to organ donation and transplantation. Its development followed a structured process that included a review of prior instruments, consultation with experts in transplantology, bioethics, and medical education, and cognitive interviews with students. The tool was pilot-tested among 30 students (not included in the study) to ensure heterogeneity in educational experience. Based on their feedback, minor adjustments were made to improve clarity and readability, including rephrasing two items concerning legal regulations and simplifying response options in one attitude-related item. Although the instrument demonstrated satisfactory face and content validity, it has not undergone formal psychometric validation, which is acknowledged as a limitation of this study.

The questionnaire was prepared in two versions: one for students of medicine and one for students of other healthcare disciplines. The basic version of the questionnaire was aimed at students in the students of other healthcare disciplines group and consisted of 16 questions, including 12 single-choice questions, 4 multiple-choice questions, and a metrics section. The version targeting the students of medicine group students was expanded to include an additional two questions.

A transplantation support index was developed for the survey, which included responses to the following questions: (1) Do you support the procurement and transplantation of organs from deceased donors?, (2) Would you agree to your organs being taken in the event of your death?, (3) Would you accept your loved one’s organs being taken after their death?, (4) Do you support the removal of organs from living donors?, (5) Is organ transplantation against your religion or ethical views? All of these questions were measured on a 5-point Likert scale where 1 meant definitely no and 5 meant definitely yes. In order to create a combined index for all questions, the order of responses to the question Is organ transplantation contrary to your religion or ethical views? This resulted in a consistent interpretation of the answers for each question, with answer 1 being the most opposed basis for transplantation and answer 5 being the maximum support for transplantation. The resulting index, which summed the answers from the questions listed above, could take values from 5 to 25, where the higher the level of the index, the more support for transplantation the person surveyed presented.

The reliability of the questionnaire was tested in a pilot study conducted among second-year public health students in the end of 2017.

### Statistical analysis

Statistical analysis was carried out using R version 3.5.1. The results of data collected during the research process were presented using basic descriptive statistics, according to the measurement scale of the individual variables. Categorical variables were presented using frequency measures: the number n and the percentage of the whole group. Quantitative variables were described using measures of central tendency and measure of dispersion. Normality of distribution of quantitative variables in subgroups was checked using Shapiro–Wilk, Kolmogorov–Smirnov tests. Equality of subgroups was checked by chi-square test. Comparison of groups of students of medicine and other majors was performed using the chi-square test (categorical variables) or non-parametric tests (Mann–Whitney *U* and Kruskal–Wallis) for quantitative variables because the assumptions of the parametric test were not met (high skewness of data, lack of normality of distribution, and lack of equality of groups). When statistically significant differences were found in the Kruskal–Wallis test, Dunn’s post-hoc test was performed to identify specific subgroups responsible for the differences found. Bonferroni correction for multiple comparisons was applied. For analyses of data on large groups of respondents, as in the case of the present study, statistical tests indicate the statistical significance of even non-significant differences between groups that may not be significant. Therefore, for a complete picture of the statistical analysis performed, a measure of the strength of the effect of the difference found was calculated for each test in addition to the significance level p testing the statistical significance of the difference between groups. Measures of the strength of the effect appropriate to the statistical tests used were used. For the chi-square test with tables of dimension 2 × 2, it was the phi coefficient (*ϕ*), while for tables of larger dimensions it was the Cramer’s *V* coefficient (V). For nonparametric tests, on the other hand, Glass’s rank bisection correlation coefficient (rg) was used with the U-Mann Whitney test or epsilon-square coefficient (ε2) for the Kruskal-Wallis test.

## Results

### Sociodemographic characteristics of the study groups

Women represented just over half of the students of medicine group (63%) and the vast majority of the students of other healthcare disciplines (90%). A statistically significant difference in sex structure between the two groups was confirmed with a significantly higher percentage of women in the students of other healthcare disciplines, but the difference was of weak strength, *ϕ* = 0.05, *p* < 0.001. Students of medicine group were of a median age of 21 years, with the youngest being 18 years old and the oldest 32 years old. In contrast, the students of other healthcare disciplines had a median age of 22 years, and the age range of this group of subjects ranged from 18 to 55 years. It was confirmed that the mean age of students of other healthcare disciplines was significantly higher than that of students of medicine, but with a weak strength of effect, rg = −0.14, *p* < 0.001. In the case of place of residence before entering the study, students from the students of medicine group most often indicated cities with population over 200 thousand (32%), while the students of other healthcare disciplines group most often indicated a village as their place of residence (33%). Both groups differed significantly in the structure of answers in the question about place of residence with a strong effect, *V* = 0.18, *p* < 0.001. The students of other healthcare disciplines significantly more often lived in the countryside (33% vs. 19% for students from the students of medicine group, *p* < 0.001), while students from the students of medicine group more often lived in large cities (50–100 thousand inhabitants, 100–200 thousand inhabitants, and larger). Taking into account the education of both parents, the respondents of the groups differed in a statistically significant manner with a very strong difference for both mother’s (*V* = 0.31, *p* < 0.001) and father’s (*V* = 0.38, *p* < 0.001) education. The students of medicine group was dominated by indications of higher education (both mother—75% of respondents and father—66%, compared to 41% and 26%, respectively, for the students of other healthcare disciplines group). In the students of other healthcare disciplines group, secondary education was most frequently indicated (46% for mother’s education and 56% for father’s education). Respondents in the students of other healthcare disciplines group were significantly more likely to have parents with primary (*p* < 0.001 for both father’s and mother’s education) and secondary (*p* < 0.001 for both parents) education than students in the students of medicine group. Students in the students of medicine group were significantly more likely to have both parents with a college education (*p* < 0.001 for both parents). Both groups of respondents indicated most often that they had a satisfactory material situation (44% from the students of medicine group and 52% of students from the students of other healthcare disciplines group). A statistically significant difference between both groups in the structure of answers about the material situation with a strong effect was confirmed (*V* = 0.15, *p* < 0.001). Students in the students of medicine group significantly more often than students in the students of other healthcare disciplines group answered that they had a bad material situation (*p* < 0.001), while students in the students of other healthcare disciplines group significantly more often indicated a satisfactory (*p* < 0.001) or good (*p* = 0.007) material situation.

### Students attitudes towards transplantation

Analysis of the value of the calculated index of transplantation support, in the entire study group of students showed that it had a median response of 23.00 (IQR: 21.00–24.00) with a maximum possible value of 25. This overall score reflects generally strong pro-donation attitudes among students, yet notable differences emerged between the two study groups. Among medical students, the median Transplantation Support Index (TSI) was 24.00 (IQR: 21.00–25.00; mean ± SD: 23.41 ± 3.02), while among students of other healthcare disciplines it was 22.00 (IQR: 20.00–24.00; mean ± SD: 21.87 ± 3.45; *p* < 0.001). The analysis of the structure of answers to individual questions for the entire study group shows that the highest number of answers supporting transplantation (answers 4—yes and 5—definitely yes) was given to the question about supporting transplantation from deceased donors (97% of respondents) and to the question about taking the respondent’s own organs after death (91%). The remaining questions received supportive response rates of 80%–82%. It is worth mentioning the double-digit percentage of respondents who could not specify their preference (answer 3-difficult to say) for the questions concerning: transplantation from a close relative, obtaining organs from living donors, and knowledge concerning compatibility of transplantation with the religion. The percentage of undecided respondents was lowest for the question about support for transplantation from deceased donors (2%). Responses indicating a position against transplantation (responses of 1—definitely no and 2—no) accounted for 1%–6%, depending on the question (1% for the question on support of transplantation from deceased donors, and 6% in the question on living donor organ procurement). Taken together, these results indicate that while nearly all students support organ donation in principle, uncertainty remains around living and family-related donation, suggesting that ethical and emotional aspects of transplantation evoke greater hesitation.

For students of medicine, support for transplantation (answers 4—yes and 5—definitely yes) was also highest for aspects related to the procurement of organs from deceased donors (97%) and consenting to the procurement of respondents’ own organs after death (93%). For the remaining questions, support ranged from 86% to 89% depending on the question. The percentage of those opposed to transplantation (answers 1—definitely no and 2—no) was lowest for the question on procurement of organs from deceased donors (2%), while for the other questions it was 3%–5%, depending on the question. The percentage of undecided was lowest for the question on organ procurement from deceased donors (1%), while it was highest for the question on consent for organ procurement from a loved one (9%) and the question on organ procurement from living donors (10%). Overall, medical students presented a more consistent and affirmative stance across all domains of transplantation, reflecting both higher confidence and lower indecision compared with their peers from other healthcare disciplines.

In the students of other healthcare disciplines, the highest support for transplantation (answers 4—yes and 5—definitely yes) concerned—similarly as in the students of medicine-taking organs from deceased persons (96%) and consent for own organs after death (90%). Other aspects, i.e., questions about transplantation from a loved one, organ procurement from living donors, and knowledge regarding compatibility of transplantation with one’s religion, received 78%–79% support. The lower support in these areas is primarily related to the presence of those unable to state an opinion (answer 3—hard to say), 15%–17% of respondents, depending on the question (compared to 7%–10% for the students of medicine). The percentage of those opposed to transplantation (answers 1—definitely not and 2—no) was 1%–7% depending on the question (compared to 2%–5% for the students of medicine). The highest number of responses against transplantation concerned aspects of organ procurement from living donors (7%), while the lowest number concerned organ procurement from deceased persons (1%). The contrast between groups was most pronounced in questions requiring personal or relational commitment, such as consenting to donation from a loved one or evaluating the compatibility of transplantation with ethical or religious beliefs where indecision among non-medical students reached nearly twice that observed among medical students.

Next, the level of the transplantation support index and its components were compared between the groups of students of medicine and other healthcare disciplines. A statistically significant difference in the mean level of the transplantation index between both groups of students was confirmed, but of weak strength, rg from 0.09 to 0.17, *p* < 0.001. Students of medicine had significantly higher levels of support than students of other healthcare disciplines (median = 24.00 and median = 22.00, respectively). The difference in overall transplantation support between both groups was statistically significant but of weak strength (rg = 0.20, *p* < 0.001). Importantly, the pattern was consistent across all component questions, confirming that medical students not only express stronger support but also demonstrate a more consolidated attitude toward transplantation ethics and practice.

The analysis also includes verification of the hypothesis that the percentage of undecided persons differs between students of medicine and students of other healthcare disciplines. For this purpose, the percentage of those who gave a 3—hard to say answer to each question included in the transplantation support index was compared between groups. It was confirmed that for each question the percentage of undecided was significantly higher in the students of other healthcare disciplines than in the students of medicine (*p* < 0.001 for each question). The power of the effect of the confirmed difference was highest for the questions: Do you accept the possibility of organ donation from a loved one after death? and Is organ transplantation compatible with your religion or ethical views? and had a moderate level.

### Knowledge of legal aspects of organ transplantation

In the study group, almost half of the respondents were able to correctly identify the legal requirements for consent for organ procurement (49%), with 66% in the students of medicine group and 41% of respondents in the students of other healthcare disciplines group. The wrong answer was given by 45% of respondents, while 7% of respondents admitted that they did not know the legal regulations related to transplantation. Students who incorrectly answered the question about legal regulations connected with transplantation, most often answered that in order to carry out transplantation, the consent of the donor given during his/her lifetime was necessary (23% of the entire group), or the consent of the family of the deceased (20%). A marginal percentage of respondents (2%) answered that there were no legal regulations concerning transplantation in Poland. It was confirmed that students of medicine differed statistically significantly from students in other majors in terms of their knowledge of transplantation regulations, with a strong power of the effect of the difference indicated (*V* = 0.23, *p* < 0.001). Students of medicine group were significantly more likely to know the law related to transplantation (*p* < 0.001), while students of other healthcare disciplines group were significantly more likely than those in the students of medicine group to incorrectly indicate that in order to harvest organs from the deceased, the deceased’s consent was needed while they were alive (*p* < 0.001) or the consent of the deceased’s family (*p* < 0.001).

### Sources of knowledge about organ transplantation

Respondents most often indicated that they get their knowledge about transplantation from college classes (63%) and the Internet (62%), slightly less often from television (36%). The comparison of the frequency of indicating particular channels of acquiring knowledge about organ transplantation between the students of medicine and students of other healthcare disciplines groups indicates that the representatives of the latter group significantly more often indicated television (*p* < 0.001) and the Internet (*p* < 0.001). For both channels, however, the strength of the effect of the difference between the two groups was weak (*ϕ* = 0.07 and ϕ = 0.08, respectively). The frequency of indicating the other knowledge transfer channels considered was not significantly different between the two groups.

More than half of the respondents (58%) have encountered a social campaign on organ transplantation, 27% gave a negative answer. The remaining 15% of respondents were unable to say whether they had encountered a similar campaign. A statistically significant difference was confirmed between the students of medicine and students of other healthcare disciplines groups in terms of frequency of contact with the organ transplantation public awareness campaign, but with weak strength of effect (*V* = 0.07, *p* < 0.001). Half of the respondents in the study group (52%) indicated that they had classes related to transplantation medicine during their studies, and 38% of the respondents gave a negative answer in the considered question. The remaining 10% of the respondents did not remember whether the topic of organ transplantation was covered in their study program. A statistically significant difference was confirmed between the students of medicine and students of other healthcare disciplines groups in the percentage of indications that transplantation medicine classes were present during their studies, with a moderate strength of effect, *V* = 0.10, *p* < 0.001. Students of other healthcare disciplines were significantly more likely to indicate that they had transplantation-related classes during their studies than students of medicine group (*p* = 0.002, 54 and 47% of respondents from both groups, respectively).

## Discussion

Despite significant progress over the past decades, the list of patients waiting for transplants is very long, and the gap between organs harvested and demand continues to widen. This study examined the attitudes of students of medicine in Poland toward organ procurement and transplantation. The participants of the study as future employees of the health care sector, will be able to participate directly or indirectly in the process of organ procurement and transplantation. Shaping pro-transplantation attitudes especially in the groups of future health care professionals is currently one of the chances for increasing the number of transplants and thus saving lives and restoring health.

### Students attitudes towards transplantation

Results obtained in own research confirm a general trend observed in support for transplantation in Poland. Regular surveys carried out by CBOS show that 93% of Poles are favorable to the idea of transplantation, 80% of Poles agree for taking organs after death and 11% are against it (23). In a study by H. Sahin and O. Abbasoglu of 1,541 students of medicine from 104 countries, 94.4% said they supported organ transplantation, 4.3% chose do not know, and 1.4% opposed the idea of donation (24). A high level of acceptance for organ transplantation (99.6%; *n* = 273) was indicated in a study among students of the Medical University of Bialystok by Rydzewska-Rosołowska et al. ((25)). Transplantation of organs to living donors was supported by 98.9% of students, and 97.1% of students supported transplantation of organs from deceased donors. In a survey of 558 students of medicine at the Aristotle University of Thessaloniki, 93.9% of respondents supported the idea of organ donation, but nearly 80% of Greek students were not aware of the legal regulations in this area (26). Similarly, recent research conducted in Finland in 2025 confirmed strong support for organ donation among students of medicine (97.6%) and nursing (94.5%). Comparable levels of global support coupled with uneven legal literacy and variable readiness to discuss donation with families have been documented in recent studies in Canada, Turkey, Brazil, Saudi Arabia, and Hong Kong, suggesting a broadly shared pattern across diverse legal and cultural settings (17–22). This international confirmation of positive attitudes aligns closely with the findings of the present study and highlights that healthcare students, regardless of country, represent a highly receptive group for transplantation-related education and promotion (27).

### Knowledge of legal aspects of organ transplantation

Our own research also revealed gaps in students’ knowledge of current legislative solutions. In the entire study group, nearly 49% of students correctly indicated the current model of regulation, respectively 66% for the students of medicine and 41% for the students of other healthcare disciplines group. It is disturbing that as many as 45% of the students gave an incorrect answer, and 7% admitted to having no knowledge on the subject. The students of medicine group was significantly more likely to indicate the correct answer. In contrast, the students of other healthcare disciplines group was significantly more likely than students of medicine group to indicate the non-mandatory model of explicit consent or necessary family consent. These findings are consistent with results from Portuguese universities, where Costa Silva et al. (28) found that although 92% of medical students correctly identified the “opt-out” legal model, less than 40% could name basic immunosuppressive drugs used after transplantation. This indicates that even in countries where legal knowledge appears sufficient, essential clinical knowledge may still be lacking. Thus, the issue is not only legal literacy but also the breadth and depth of content covered in education programs.

These disparities are not merely individual but stem from structural and curricular factors. In many Polish universities, legal education related to transplantation is fragmented, often integrated into ethics or health policy courses without explicit learning outcomes or assessment. Non-medical programs such as nursing, midwifery, and public health rarely put an emphasis on a distinct component on transplantation law, even though these professionals frequently interact with families and patients in clinical settings. This curricular gap may explain why 20% of our respondents incorrectly believed that family consent is required and 23% thought that explicit donor consent during lifetime is necessary. Such misconceptions likely reflect deficiencies in procedural and communication training rather than theoretical understanding, suggesting the need for systemic rather than *ad hoc* educational reforms. Moving beyond the general call for “more education,” a concrete and standardized framework should be developed. One possible solution would be to implement a short, mandatory micro-module on transplantation law and professional responsibility (approximately 6–8 h) for all healthcare programs, ideally integrated into the second or third year of study. This module could include scenario-based assessments and simulation exercises testing students’ ability to communicate with families and verify objections in the Central Register. Complementary integration of transplantation topics into ethics and clinical communication courses would further strengthen learning transfer. Measurable indicators such as a minimum 80% pass rate on legal knowledge tests and periodic curriculum audits could ensure long-term consistency across universities. As shown by Abbasi et al. (29), legal education that combines lectures, case analysis, and assessments has been effective in improving medical students’ cognitive domains in medico-legal knowledge. Similarly, Chen et al. (30) demonstrated that a case-based curriculum integrating legal and ethical scenarios enhanced students’ confidence, communication with patients and families, and understanding of the legal dimension of clinical practice.

In comparative terms, Poland’s presumed consent (opt-out) model aligns legally with countries such as Spain and Austria, yet the observed donation rates and knowledge levels remain substantially lower. This discrepancy supports previous findings that legislative models alone do not determine donation outcomes. In Spain, the long-standing opt-out framework is reinforced by intensive public education, clinical coordination, and social normalization of donation (7, 12). Similarly, Austria maintains high donation rates through strong institutional infrastructure and consistent communication between healthcare providers and the public. Conversely, countries with opt-in systems, such as Germany or Turkey, continue to face barriers related to family consent, limited awareness, and sociocultural hesitation despite widespread support in principle (10, 24). The influence of cultural and religious beliefs on donation attitudes has also been confirmed in other academic settings. A recent studies among students in Turkey revealed that while participants generally expressed positive views toward blood donation, their willingness to donate organs was considerably constrained by knowledge gaps, religious concerns, and personal apprehensions (31, 32). These cross-national contrasts indicate that universal challenges, such as emotional and ethical ambivalence or insufficient legal literacy manifest differently depending on national contexts and educational traditions, emphasizing the importance of comprehensive, context-sensitive educational strategies. A particularly noteworthy finding is the comparatively large proportion of “undecided” respondents among non-medical students, reaching up to 17% in questions concerning living or family-related donation. This group likely represents individuals with ambivalent or insufficiently formed attitudes, potentially reflecting a lack of knowledge, limited exposure to clinical contexts, or uncertainty arising from religious and ethical considerations. Similar patterns have been described in studies from Turkey and Poland, where hesitation toward organ donation was associated with perceived moral conflict and low confidence in understanding legal or medical procedures (31–33). From a public health perspective, this “undecided” cohort may constitute the most malleable target group for educational interventions, as their views appear not firmly opposed but rather shaped by informational deficits and emotional reservations. Future studies should explore this group qualitatively to better understand the cognitive and affective factors underlying indecision and to guide targeted communication strategies.

Beyond the dichotomy of opt in and opt out systems, recent scholarship has emphasized deeper ethical and structural challenges within organ donation frameworks. Ambagtsheer et al. (2024) highlight emerging debates concerning donor anonymity in kidney exchange programs, the inclusion of individuals with limited decision making capacity, and the ethical legitimacy of compensation mechanisms (34). These factors demonstrate that societal trust and transparency remain central to sustaining donation systems. Similarly, Lewis et al. (35) argue that a comprehensive understanding of organ donation requires integrating legal literacy with ethical reasoning, especially regarding autonomy, informed consent, and justice. Addressing these dimensions through academic education is essential to prepare healthcare students for the moral complexity of transplantation practice.

In the survey conducted by CBOS, the model of consent given during life has a similar number of supporters as the one in which the absence of objection is sufficient. In 2016, 43% of Poles opted for the explicit consent model and 42% for the implicit consent model. Comparing the 2012 survey, it should be noted that respondents were less likely to choose the explicit consent model (23, 36). Compared to the nationwide study, in which in 2016 20% of respondents correctly answered, the students surveyed definitely more often indicated the model of implicit consent as valid, in the CBOS study the model of implicit consent was more often advocated by respondents aged 25–34 years. It can be assumed that this solution is more often chosen by younger age groups (23). It is worth emphasizing that knowledge of current legislation in this area can affect the process of organ transplantation. A nationwide survey shows that Poles either have no knowledge of this topic at all or indicate an incorrect answer.

The information provided by medical personnel is crucial in creating a social climate around the topic of transplantation. This is why the knowledge gained during university education is so important. Students, as future professionals in the health sector, must provide accurate information based on sound knowledge. In addition, they should use clear concepts with which to communicate with the public in a concise manner to prevent irrational fears of organ donation (37).

### Sources of knowledge about organ transplantation

It should be noted that the most common sources from which students obtained information on organ transplantation were academic classes (62.6%), the Internet (61.8%), television (35.5%) and books (18.8%) (38, 39). The finding that the Internet represents the second most common source of information (62%) is particularly concerning, as online materials on transplantation often include unverified or outdated content. Heavy reliance on digital sources without adequate academic guidance can perpetuate myths about consent, brain death, and the legal role of families. Therefore, university-based curricula should incorporate elements of critical information appraisal, such as short workshops or assignments focusing on evaluating online sources and distinguishing between official legal documents (e.g., the Transplantation Act, Poltransplant reports) and informal media narratives. Strengthening digital literacy in this domain is essential to reduce misinformation and enhance evidence-based communication with patients and the public.

It is worth mentioning that half of the students from the entire study group indicated that they had classes on transplantation issues during their studies, nearly 40% denied having such classes, and 10% did not remember whether this topic was covered in their academic classes. A comparison between the students of medicine and students of other healthcare disciplines groups showed that students from other majors were more likely to indicate that they had had classes on the topic discussed than students of medicine. This may be due to the fact that transplantation issues are covered in different subjects and students were not able to identify this as one subject throughout their studies.

As H. Sahin and O. Abbasoglu point out, in order to provide future physicians with the necessary knowledge and equip them with appropriate attitudes regarding organ donation, an appropriate educational strategy is necessary. The curricula of many medical universities do not give adequate emphasis to this topic. Determining students’ knowledge about organ donation is considered the first step to developing such an educational model. In the study cited above, participants who received a course in organ donation showed higher scores than participants who did not receive such education (18). Darlington et al. (40) in their work also emphasize the important role of holding regular seminars for medical students on organ procurement and transplantation.

In a recent Polish study by Mikla et al. (33) similar deficits in formal education were identified. The authors emphasized the role of family discussions and social campaigns in shaping attitudes and knowledge among students. Notably, the absence of family conversations on the subject correlated with less clarity regarding the legal framework. These observations support the thesis that academic teaching should not only provide factual knowledge but also encourage intergenerational dialogue and ethical reflection.

This study shows that the participants represent a group of future health care professionals directly or indirectly influencing the process of organ donation. Therefore, proper education of this group is extremely important, as it can significantly affect the number of transplants performed.

As emphasized by Radunz et al. (41) health care professionals should be the most knowledgeable group in the field of organ donation. The problem of organ shortage is a health care problem and can be solved by a strong attitude of physicians and other health care personnel. Early education of health care professionals thus appears to be a factor that can serve to maximize the benefits of a limited donor pool.

It should still be taken into account that studying is a process during which attitudes and beliefs towards many social phenomena are formed among young people including organ procurement and transplantation, and due care must be taken to equip them with pro-transplantation attitudes (42, 43). As indicated by the European Commission, students have been identified as one of the most helpful social groups among the population of the European Union for the formation of positive attitudes towards organ procurement and transplantation (44). It is emphasized that students represent a high level of altruistic social commitment and have a strong influence on their family members, friends, acquaintances, neighbors (45).

### National context in Poland

Our results are in line with contemporary Polish evidence showing that medical students generally declare strong support for transplantation, yet decisions regarding donation on behalf of relatives are strongly influenced by family discussions, a sense of moral duty, and solidarity, while the absence of such conversations is associated with reduced willingness to donate (33). Single university studies from Bialystok confirm nearly universal acceptance of transplantation but also reveal gaps in the acceptance of brain death criteria, limited readiness for postmortem donation, and the decisive role of family in the donation process (25, 46). In the broader student population, beyond medical programs, positive attitudes do not consistently translate into concrete actions, with low rates of actual donor registration indicating a gap between declared support and real behavior (47). Furthermore, in the area of tissue donation such as corneal transplantation, knowledge deficits persist across different social groups in Poland, and the disparity between demand and the number of procedures performed underscores the importance of targeted education and public campaigns (48). Collectively, these findings reinforce our recommendation for systematic, standardized, and multidisciplinary education on transplantation across all health related academic programs. Poland’s opt-out model presents an apparent paradox: despite a presumed consent system, national donation rates remain among the lowest in the European Union. Our findings suggest that this paradox may, at least partially, originate from micro-level knowledge and communication deficits among healthcare students and professionals. When nearly one in five respondents believes that family consent is required, the legal framework of presumed consent becomes functionally undermined. In practice, uncertainty about the law may lead to unnecessary hesitation in donor identification or family discussions, effectively reintroducing a “family veto” into a system where it does not legally exist. Addressing these misconceptions through structured education could therefore have macro-level implications, potentially improving the efficiency of the opt-out system and increasing actual donation rates.

### Practical implications

Our findings highlight the urgent need to reinforce educational strategies in the field of organ donation and transplantation. Previous studies confirm that targeted and structured educational programs significantly improve medical students’ knowledge and acceptance of transplantation (49, 50). Moreover, online and institution-based interventions have been shown to increase willingness to donate organs among university communities, demonstrating that well-designed educational activities can influence not only knowledge but also attitudes and intentions (51). Given the heavy reliance on informal online sources observed in this study, structured academic interventions are essential to ensure that future healthcare professionals base their knowledge on verified and legally accurate information rather than social media or non-specialist websites. Therefore, the integration of dedicated modules into medical curricula, combined with interactive and accessible educational formats, may provide a comprehensive framework for equipping future healthcare professionals with the necessary competencies to promote organ donation.

### Limitations of the study

This study has several limitations. A major methodological limitation of this study is that the questionnaire and the Transplantation Support Index (TSI) were not subjected to a formal psychometric validation process. While the questionnaire was developed with expert input and pilot-tested to ensure clarity, it lacks established measures of content validity, construct validity, and internal consistency. Consequently, the reliability of individual subscales and the interpretability of composite scores may be limited. The TSI should therefore be regarded as an exploratory indicator, intended to provide a preliminary framework for assessing students’ overall support for organ transplantation. Future research should focus on developing and validating a standardized instrument based on this initial version. The study was conducted exclusively among students from four Polish medical universities, which may limit the generalizability of findings to other institutions or countries. Moreover, differences in curricula between universities, as well as potential gaps in the coverage of transplantation-related issues, could have influenced students’ responses and thus affected the comparability of results. The use of a self-reported questionnaire could have introduced social desirability bias, particularly in responses related to attitudes and ethical beliefs. Additionally, the data collection spanned from 2017 to 2019, a period during which educational curricula and public discourse surrounding transplantation may have evolved. Therefore, temporal variations in students’ exposure to transplantation-related content cannot be ruled out. The cross-sectional design also precludes any causal inferences regarding the relationship between educational exposure, knowledge, and attitudes. Another methodological limitation is the absence of multivariate modeling. Because the study database was fully anonymized and aggregated by subgroups, it was not possible to perform logistic or ordinal regression analyses to identify independent predictors of high transplantation support or correct legal knowledge. As a result, potential confounding effects between sociodemographic and educational variables could not be controlled for. Future research based on this instrument should prospectively collect individual-level data to allow for comprehensive multivariate analysis.

## Conclusion

The vast majority of medical school students in Poland support organ transplantation. Medical school students identified academic classes as the most common source for learning about organ transplantation. Therefore, it is necessary to introduce a multidisciplinary educational strategy in academic teaching, taking into account the medical, legal, ethical and social aspects of organ procurement and transplantation, in order to eliminate barriers to organ donation. Such a strategy should be more precisely defined and include three essential components: (1) the implementation of a standardized and mandatory legal curriculum across all healthcare faculties to ensure consistent understanding of the Polish opt-out system; (2) structured communication skills training to prepare students for discussions about organ donation with patients and families; and (3) educational initiatives aimed at dispelling persistent myths regarding brain death and clarifying the limited role of family consent under the presumed consent model. The significant difference in knowledge of legal regulations between medical students and those of other healthcare disciplines indicates the need to standardize the scope of transplantation-related education across all health-related university programs in Poland. Future research should include longitudinal studies to assess how students’ knowledge and attitudes evolve during their education, as well as comparative analyses across different universities and healthcare disciplines. In addition, the evaluation of targeted educational interventions would provide valuable evidence on the most effective strategies for promoting pro-transplantation attitudes among future healthcare professionals.

## Data Availability

The raw data supporting the conclusions of this article will be made available by the authors, without undue reservation.
